# Supplementary material for: A spectral theory for Wright’s inbreeding coefficients and related quantities
Source: PLoS Genet. 2021 Jul 19;17(7):e1009665. doi: 10.1371/journal.pgen.1009665 (PMC8320931; doi:10.1371/journal.pgen.1009665)
Supplement: S1 Text — (PDF) [file pgen.1009665.s001.pdf]

Supplementary Information for "A Spectral Theory for Wright's Inbreeding Coefficients and Related Quantities"

Olivier François Clément Gain

Université Grenoble-Alpes, Centre National de la Recherche Scientifique, Grenoble INP, TIMC-IMAG CNRS UMR 5525, 38000 Grenoble, France.

Corresponding author: olivier.francois@univ-grenoble-alpes.fr

Contents

1 Inbreeding coefficients and matrix norms **2**

1.1 Results for D_{ST} and F_{ST} 2

1.2 Details on matrix norms 3

2 Main results **3**

2.1 Proof of Theorem 1 3

2.2 Proof of Corollary 5

2.3 Approximation of D_{ST} and F_{ST} by PCA eigenvalues 6

3 Mathematical analysis of F-models **9**

3.1 F-models 9

3.2 Expected values 10

3.3 Three-population models 11

1 Inbreeding coefficients and matrix norms

1.1 Results for D_{ST} and F_{ST}

Consider K discrete populations and a sample of n organisms from those populations, where n_k individuals originate from population k. Obviously, we have $n_1 + \cdots + n_K = n$. The coefficient $c_k = n_k/n$ represents the proportion of individuals sampled from population k. Genetic variation is observed at a diallelic locus having a reference allele and a derived allele. The frequency of the derived allele in population k is equal to p_k. The frequency of the reference allele in population k is equal to $q_k = 1 - p_k$. The frequency of the derived allele in the total sample is then equal to $P = \sum_{k=1}^{K} c_k p_k$. The total heterozygozity is defined as $H_{\mathrm{T}} = 2P(1-P)$, and we set $H_{\mathrm{S}} = 2\sum_{k=1}^{K} c_k p_k q_k$. Using notations introduced in [1, 2, 3], D_{ST} is defined as the difference $D_{\mathrm{ST}} = H_{\mathrm{T}} - H_{\mathrm{S}}$, and is equal to

$$D_{\mathrm{ST}} = 2\left(\sum_{j=1}^{K}\sum_{k=1}^{K} c_j c_k p_j q_k - \sum_{j=1}^{K} c_j p_j q_j\right) .$$

Rearranging terms on the left-hand side of the above equation, we find that

$$D_{\mathrm{ST}} = 2\left(\sum_{j=1}^{K} c_j p_j \sum_{k=1}^{K} c_k (p_j - p_k)\right) = 2\sum_{j<k} c_j c_k (p_j - p_k)^2 .$$

Now, developing the expression

$$A = \sum_{k=1}^{K} c_k \left(\sum_{j=1}^{K} c_j (p_j - p_k)\right)^2 ,$$

we find that D_{ST} is also equal to

$$D_{\mathrm{ST}} = \sum_{k=1}^{K} c_k \left(\sum_{j=1}^{K} c_j (p_j - p_k)\right)^2 .$$

1.2 Details on matrix norms

For any matrix, $\mathbf{Y}$, of dimension $n \times L$, the Hilbert-Schmidt norm, or Frobenius norm, of $\mathbf{Y}$ is defined by

$$\|\mathbf{Y}\| = \left(\sum_{i=1}^{n} \sum_{\ell=1}^{L} y_{i\ell}^2 \right)^{1/2} .$$

In particular, the squared norm of $\mathbf{Y}$ corresponds to the sum of the eigenvalues of $\mathbf{Y}^T\mathbf{Y}$

$$\|\mathbf{Y}\|^2 = \mathbf{Tr}(\mathbf{Y}^T\mathbf{Y}) = n \sum_{i=1}^{\min(n,L)} \sigma_i^2(\mathbf{Y}) \, ,$$

where $\sigma_i(\mathbf{Y})$ is a non-null singular value of $\mathbf{Y}/\sqrt{n}$, and $\mathbf{Tr}(\mathbf{M})$ denotes the trace of the matrix $\mathbf{M}$. For all i, $\sigma_i^2(\mathbf{Y})$ is a non-null eigenvalue of $\mathbf{Y}\mathbf{Y}^T/n$ and of $\mathbf{Y}^T\mathbf{Y}/n$. We shall use the term *eigenvalue* as a synonymous of *squared singular value.*

2 Main results

2.1 Proof of Theorem 1

Let $\mathbf{X} = (x_{i\ell})$ record genotypes at L loci for n individuals sampled from K discrete populations. For haploids, we have $x_{i\ell} = 0, 1$, and for diploids, we have $x_{i\ell} = 0, 1, 2$. We consider autosomes, and we suppose that a diploid genome is formed of two haploid genomes. When the phase of a diploid genotype is unknown, we use an arbitrary phase, either defined as $(0, 1)$ or as $(1, 0)$, in order to create two haploid genotypes. With this simplification, the diploid case amounts to consider a sample of $2n$ haploid individuals. Let $\mathbf{Z}^{\mathrm{c}} = \mathbf{X} - \mathbf{P}$ be the centered matrix, for which the mean value of a column is null. Let $\mathbf{Z}^{\mathrm{sc}}$ be the scaled matrix in which each column has variance is equal to one, that is, $\mathbf{z}_{i\ell}^{\mathrm{sc}} = \mathbf{z}_{i\ell}^{\mathrm{c}}/\sqrt{P_\ell(1 - P_\ell)}$ [4]. For a clearer presentation,

we only describe results for $\mathbf{Z}^{\mathrm{c}}$ and omit the superscript. The same principles and results also apply to $\mathbf{Z}^{\mathrm{sc}}$ in a straightforward way.

The $n \times L$ matrix $\mathbf{Z}$ can be decomposed as the sum of between and within-population matrices, $\mathbf{Z} = \mathbf{Z}_{\mathrm{ST}} + \mathbf{Z}_{\mathrm{S}}$, as follows. For an individual i sampled from population k and an arbitrary locus ℓ, the between-population matrix $\mathbf{Z}_{\mathrm{ST}}$ has general term

$$z_{i\ell}^{st} = \sum_{j=1}^{K} c_j (p_{k\ell} - p_{j\ell}) \, ,$$

where this term is repeated n_k times at each locus, for all i in population k. The allele frequencies, $p_{k\ell}$, are specific to each locus. The within-population matrix $\mathbf{Z}_{\mathrm{S}}$ has general term

$$z_{i\ell}^{s} = x_{i\ell} - p_{k\ell} \, .$$

Here we compute the matrix norms of the between and within-population matrices separately. For the between-population matrix, we have

$$\|\mathbf{Z}_{\mathrm{ST}}\|^2 = \sum_{i=1}^{n} \sum_{\ell=1}^{L} (z_{i\ell}^{st})^2 \, ,$$

which is equal to

$$\|\mathbf{Z}_{\mathrm{ST}}\|^2 = nL \, \mathbb{E} \left[\sum_{k=1}^{K} c_k \left(\sum_{j=1}^{K} c_j (p_j - p_k) \right)^2 \right] = nL \, \mathbb{E}[D_{\mathrm{ST}}]/2 \, .$$

We use the notation $\mathbb{E}[Q] = \sum_{\ell=1}^{L} Q_\ell / L$ everywhere to denote the average value of Q_ℓ over all loci. For the within-population matrix, we have

$$\|\mathbf{Z}_{\mathrm{S}}\|^2 = \sum_{i=1}^{n} \sum_{\ell=1}^{L} (z_{i\ell}^{s})^2 \, ,$$

which is equal to

$$\|\mathbf{Z}_{\rm S}\|^2 = nL\,\mathbb{E}\left[\sum_{k=1}^{K} c_k {\rm Var}(x_i | i \in {\rm pop}_k)\right] = nL\,\mathbb{E}[H_{\rm S}]/2\,.$$

The scaled matrix, $\mathbf{Z}^{\rm sc}$, can be obtained from $\mathbf{Z}$ by dividing each column by $\sqrt{P_\ell(1-P_\ell)}$. Thus the squared norm of $\mathbf{Z}^{\rm sc}_{\rm ST}$ is equal to

$$\|\mathbf{Z}^{\rm sc}_{\rm ST}\|^2 = nL\,\mathbb{E}[F_{\rm ST}]\,.$$

This important result redefines the average value $\mathbb{E}[F_{\rm ST}]$ from the spectrum of the between-population matrix. The definition is valid regardless of sample size, number of loci and separation of between and within-components.

2.2 Proof of Corollary

Consider a matrix of L haploid genotypes for n individuals, $\mathbf{X}$. Centered PCA computes the eigenvalues and eigenvectors of the empirical covariance matrix, $\mathbf{Z}\mathbf{Z}^T/n$. (In data analyses, we use $(n-1)$ instead of n, in order to manipulate unbiased estimates of covariance matrices. Since $n \approx n-1$ for large sample sizes, taking n introduces a small bias but enable a lighter presentation.) The eigenvalues of the empirical covariance matrix correspond to the squared non-null singular values of $\mathbf{Z}/\sqrt{n}$ [5]. As in the above section, the matrix $\mathbf{Z}$ is decomposed into a sum of between and within-population components, $\mathbf{Z} = \mathbf{Z}_{\rm ST} + \mathbf{Z}_{\rm S}$. The matrix $\mathbf{Z}_{\rm ST}$ has $(K-1)$ non-null singular values. First, we note that the matrices $\mathbf{Z}_{\rm ST}$ and $\mathbf{Z}_{\rm S}$ satisfy the following orthogonality condition,

$$\mathbf{Z}_{\rm ST}^T \mathbf{Z}_{\rm S} = \mathbf{0}\,.$$

The orthogonality condition can be checked directly, by computing the product matrix coefficients for all ℓ, m in $1, \ldots, L$. Those coefficients are equal to

$$\sum_{i=1}^{n} z_{i\ell}^{st} z_{im}^{s} = n \sum_{k=1}^{K} c_k \left(\sum_{j=1}^{K} c_j (p_{k\ell} - p_{j\ell}) \right) \sum_{i \in \mathrm{pop_k}} (x_{im} - p_{km}) \,.$$

Remarking that we have

$$\sum_{i \in \mathrm{pop_k}} (x_{im} - p_{km}) = 0 \,,$$

for each k, the orthogonality condition is obviously verified.

Using the definition of the squared norm as the trace of a matrix, we now have a Pythagorean relationship for between and within-population matrices

$$\|\mathbf{Z}_{\mathrm{ST}} + \mathbf{Z}_{\mathrm{S}}\|^2 = \mathbf{Tr}(\mathbf{Z}_{\mathrm{ST}}^T \mathbf{Z}_{\mathrm{ST}}) + \mathbf{Tr}(\mathbf{Z}_{\mathrm{S}}^T \mathbf{Z}_{\mathrm{S}}) = \|\mathbf{Z}_{\mathrm{ST}}\|^2 + \|\mathbf{Z}_{\mathrm{S}}\|^2 \,.$$

This result concludes the proof of the corollary.

2.3 Approximation of D_{ST} and F_{ST} by PCA eigenvalues

In this section, we provide arguments for the following result. Assume that 1) the smallest non-null singular value of $\mathbf{Z}_{\mathrm{ST}}$ is larger than the leading eigenvalue of $\mathbf{Z}_{\mathrm{S}}$, 2) following RMT, the leading eigenvalue of $\mathbf{Z}_{\mathrm{S}}/\sqrt{n}$ is of order $(1/\sqrt{n} + 1/\sqrt{L})^2$. Then, for any non-null singular value, $\sigma_k(\mathbf{Z}_{\mathrm{ST}})$, of $\mathbf{Z}_{\mathrm{ST}}/\sqrt{n}$, there exists a singular value, $\sigma_k(\mathbf{Z})$, of $\mathbf{Z}/\sqrt{n}$ such that we have

$$|\sigma_k^2(\mathbf{Z})/L - \sigma_k^2(\mathbf{Z}_{\mathrm{ST}})/L| \leq C/L \,, \quad k = 1, \ldots, K-1 \,.$$

For $\sigma = \sigma(\mathbf{Z}_{\mathrm{ST}})$, a non-null singular value of $\mathbf{Z}_{\mathrm{ST}}/\sqrt{n}$, and $\tilde{\sigma} = \tilde{\sigma}(\mathbf{Z}_{\mathrm{S}})$, a non-null singular value of $\mathbf{Z}_{\mathrm{S}}/\sqrt{n}$, the left singular vectors, $\mathbf{u}$ and $\tilde{\mathbf{u}}$, associated with σ and $\tilde{\sigma}$ are orthogonal vectors. To see it, compute

$$n^2 \sigma^2 \tilde{\sigma}^2 \mathbf{u}^T \tilde{\mathbf{u}} = \mathbf{u}^T \mathbf{Z}_{\mathrm{ST}} (\mathbf{Z}_{\mathrm{ST}}^T \mathbf{Z}_{\mathrm{S}}) \mathbf{Z}_{\mathrm{S}}^T \tilde{\mathbf{u}}.$$

Since $\mathbf{Z}_{\mathrm{ST}}^T \mathbf{Z}_{\mathrm{S}} = \mathbf{0}$, the middle term is null and the left singular vectors, $\mathbf{u}$ and $\tilde{\mathbf{u}}$, satisfy $\mathbf{u}^T \tilde{\mathbf{u}} = 0$.

Assuming that $L/n = \gamma$ for large values of L and n, we now show that a non-null singular value of the between-population matrix, $\mathbf{Z}_{\mathrm{ST}}/\sqrt{n}$, is an approximate singular value of the genotype matrix, $\mathbf{Z}/\sqrt{n}$. A similar result holds for the eigenvalues of the residual matrix, $\mathbf{Z}_{\mathrm{S}}/\sqrt{n}$. To show the result for the between-population matrix, let $\mathbf{u}$ be a left singular vector of $\mathbf{Z}_{\mathrm{ST}}/\sqrt{n}$ and let σ be the corresponding non-null singular value. We have

$$\mathbf{Z}\mathbf{Z}^T \mathbf{u}/n = \mathbf{Z}_{\mathrm{ST}} \mathbf{Z}_{\mathrm{ST}}^T \mathbf{u}/n + \mathbf{Z}_{\mathrm{S}} \mathbf{Z}_{\mathrm{ST}}^T \mathbf{u}/n.$$

For the first term at the right-hand side, we have $\mathbf{Z}_{\mathrm{ST}} \mathbf{Z}_{\mathrm{ST}}^T \mathbf{u}/n = \sigma^2 \mathbf{u}$. For the second term, we have $\mathbf{Z}_{\mathrm{ST}}^T \mathbf{u}/\sqrt{n} = \sigma \mathbf{v}$, where $\mathbf{v}$ is a right singular vector of $\mathbf{Z}_{\mathrm{ST}}^T/\sqrt{n}$, and

$$\mathbf{Z}\mathbf{Z}^T \mathbf{u}/n = \sigma^2 \mathbf{u} + \sigma \mathbf{Z}_{\mathrm{S}} \mathbf{v}/\sqrt{n}\,.$$

By definition of the spectral radius, we have

$$\|\mathbf{Z}_{\mathrm{S}} \mathbf{v}/\sqrt{n}\|^2 \leq \mathrm{radius}(\mathbf{Z}_{\mathrm{S}} \mathbf{Z}_{\mathrm{S}}^T/n)\,.$$

Now, note that the eigenvalue σ^2 is of order $O(L)$ (we have $\sigma^2 < \|\mathbf{Z}_{\mathrm{ST}}\|^2/n = O(L)$ by Theorem 1). According to assumption 2) for $\mathbf{Z}_{\mathrm{S}}$, we have

$$\sigma^2 \|\mathbf{Z}_{\mathrm{S}} \mathbf{v}/\sqrt{n}\|^2 = (1 + \sqrt{\gamma})^2 \times O\left(1\right)\,.$$

Put together, the last equations imply that

$$\mathbf{Z}\mathbf{Z}^T \mathbf{u}/n = \sigma^2 \mathbf{u} + O(1)\,.$$

Thus, for large n and L, $\mathbf{Z}\mathbf{Z}^T \mathbf{u}/n \approx \sigma^2 \mathbf{u}$ plus a negligible term, meaning that $\mathbf{u}$ is an approximate eigenvector of $\mathbf{Z}/\sqrt{n}$, and σ^2 is an approximate eigenvalue of

$\mathbf{Z}/\sqrt{n}$. Now, we consider the vector space spanned by the first $K-1$ eigenvectors of $\mathbf{C}_{\mathrm{ST}} = \mathbf{Z}_{\mathrm{ST}}\mathbf{Z}_{\mathrm{ST}}^T/n$. Then, consider

$$\mathbf{C}_{\mathrm{ST}}\mathbf{U} = \mathbf{U}\Sigma\,,$$

where $\Sigma = \mathrm{diag}(\sigma_1^2, \ldots, \sigma_{K-1}^2)$ and $\mathbf{U} = (\mathbf{u}_1, \ldots, \mathbf{u}_{K-1})$ are the non-null eigenvalues and left singular vectors of $\mathbf{Z}_{\mathrm{ST}}/\sqrt{n}$. For $\mathbf{C} = \mathbf{Z}\mathbf{Z}^T/n$, we have

$$\mathbf{U}^T\mathbf{C}\mathbf{U} = \Sigma + \mathbf{U}^T O(1)\,.$$

The condition number of $\mathbf{U}$ being equal to one, we can apply the Bauer-Fike Theorem [7, 6]. For each non-null singular value of $\mathbf{Z}_{\mathrm{ST}}/\sqrt{n}$, $\sigma^2(\mathbf{Z}_{\mathrm{ST}})$, there is a singular value of $\mathbf{Z}/\sqrt{n}$, $\sigma^2(\mathbf{Z})$, such that

$$|\sigma^2(\mathbf{Z}) - \sigma^2(\mathbf{Z}_{\mathrm{ST}})| = O(1)\,.$$

Using the results on matrix norms established in the previous section, we have, on one hand

$$\|\mathbf{Z}_{\mathrm{ST}}\|^2 = nL\mathbb{E}[D_{\mathrm{ST}}/2],$$

while, on another hand, we have

$$\|\mathbf{Z}_{\mathrm{ST}}\|^2 = \mathbf{Tr}(\mathbf{Z}_{\mathrm{ST}}\mathbf{Z}_{\mathrm{ST}}^T) = n\sum_{k=1}^{K-1}\sigma_k^2(\mathbf{Z}_{\mathrm{ST}})\,.$$

Under the separation condition and the RMT hypothesis, we have

$$\sum_{k=1}^{K-1}\sigma_k^2(\mathbf{Z}) \approx \sum_{k=1}^{K-1}\sigma_k^2(\mathbf{Z}_{\mathrm{ST}})\,,$$

and

$$L \times \mathbb{E}[D_{\mathrm{ST}}]/2 = \sum_{k=1}^{K-1}\sigma_k^2(\mathbf{Z}_{\mathrm{ST}}) \approx \sum_{k=1}^{K-1}\sigma_k^2(\mathbf{Z})\,.$$

For centered PCA, we obtained that $L\mathbb{E}[D_{\mathrm{ST}}]/2$ is close to the sum of the $(K-1)$ leading eigenvalues of $\mathbf{Z}/\sqrt{n}$. Turning to scaled PCA creates no complication. Since the empirical correlation matrix is obtained by scaling the columns of $\mathbf{Z}$, a spectral decomposition holds for the scaled matrix as well. For scaled PCA, the sum of the $(K-1)$ leading eigenvalues is equal to

$$L \times \mathbb{E}[F_{\mathrm{ST}}] = \sum_{k=1}^{K-1} \rho_k^2(\mathbf{Z}_{\mathrm{ST}}) \approx \sum_{k=1}^{K-1} \rho_k^2(\mathbf{Z})\,.$$

In other words, $\mathbb{E}[F_{\mathrm{ST}}]$ can be approximated by the proportion of variance explained by the first $(K-1)$ principal components of the genotype matrix. The accuracy of the approximation is expected to be of order $O(K/L)$ with respect to K and L. This error term is supported by numerical simulations of F-models (Figure SI A, Figure S5 and Figure S9).

3 Mathematical analysis of F-models

3.1 F-models

F-models are discrete population models in which K populations diverged from an ancestral gene pool [8]. The models are without mutation, migration or selection. In the ancestral gene pool, the frequency of the derived allele is equal to p_{anc}. The populations diverged from each other with population-specific drift coefficients, F_k, relative to the ancestral pool. Conditional on p_{anc}, the allele frequency at a particular locus in population k follows a beta distribution of shape parameters $p_{\mathrm{anc}}(1-F_k)/F_k$ and $(1-p_{\mathrm{anc}})(1-F_k)/F_k$. To create a distribution over the L loci, p_{anc} is drawn from a beta probability density with shape parameters a and b. The expected value for this distribution is equal to $\mathbb{E}[p_{\mathrm{anc}}] = a/(a+b)$. F-models parameters are chosen

so that $\mathbb{E}[p_k|p_{\text{anc}}] = p_{\text{anc}}$ and $\text{Var}(p_k|p_{\text{anc}}) = p_{\text{anc}}(1-p_{\text{anc}})F_k$, for all k. For F-models, the expected ancestral heterozygozity is equal to

$$\mathbb{E}[H_{\text{A}}] = \int_0^1 2p_{\text{anc}}(1-p_{\text{anc}})f(p_{\text{anc}})dp_{\text{anc}} = \frac{2ab}{(a+b)(a+b+1)}\,.$$

3.2 Expected values

In this section, we compute the expected values of D_{ST}, H_{S} and H_{T} under the F-model. Those results may not be new, but they are difficult to find from the existing literature. For the first quantity, we have $D_{\text{ST}} = 2\mathbb{E}[\sum_{j<k} c_j c_k (p_j - p_k)^2]$. Given p_{anc}, the conditional expectation of the squared allele frequency difference $(p_j - p_k)^2$ is equal to

$$\mathbb{E}[(p_j - p_k)^2|p_{\text{anc}}] = \mathbb{E}[(p_j - p_{\text{anc}})^2|p_{\text{anc}}] + \mathbb{E}[(p_k - p_{\text{anc}})^2|p_{\text{anc}}]\,.$$

The sum of squares arises from the interpretation of the F-model as a starlike tree model of divergence or more pragmatically from the conditional independence of the allele frequencies p_j and p_k given p_{anc}. This leads us to

$$\mathbb{E}[(p_j - p_k)^2|p_{\text{anc}}] = p_{\text{anc}}(1-p_{\text{anc}})(F_j + F_k)\,.$$

Thus we have

$$\mathbb{E}[\sum_{j\neq k} c_j c_k (p_j - p_k)^2|p_{\text{anc}}] = p_{\text{anc}}(1-p_{\text{anc}})\sum_{j\neq k} c_j c_k (F_j + F_k)\,.$$

Reorganizing the sum, we find that

$$\sum_{j\neq k} c_j c_k F_j = \sum_{j=1}^{K} c_j F_j \sum_{k\neq j}^{K} c_k = \sum_{j=1}^{K} c_j (1-c_j) F_j\,,$$

and we can conclude that

$$\mathbb{E}[D_{\mathrm{ST}}] = \left(\sum_{k=1}^{K} c_k(1 - c_k)F_k \right) \mathbb{E}[H_{\mathrm{A}}] \, .$$

Following similar calculations, the results for H_{T} and H_{S} are given by

$$\mathbb{E}[H_{\mathrm{T}}] = \left(1 - \sum_{k=1}^{K} c_k^2 F_k \right) \mathbb{E}[H_{\mathrm{A}}] \, .$$

and

$$\mathbb{E}[H_{\mathrm{S}}] = \left(1 - \sum_{k=1}^{K} c_k F_k \right) \mathbb{E}[H_{\mathrm{A}}] \, ,$$

We did not obtain an explicit analytical formula for the expected value of F_{ST} under the F-model. For computing $\mathbb{E}[F_{\mathrm{ST}}]$, we relied on numerical integration performed by a Monte-Carlo method.

3.3 Three-population models

For three-population F-models with equal sample sizes and for ancestral allele frequencies distributed according to the uniform distribution, beta($a = 1$, $b = 1$), the 3×3 matrix Λ corresponding to the between-population covariance matrix for allele frequencies can be calculated as

$$\mathbf{\Lambda} = \frac{1}{162} \begin{pmatrix} 4F_1 + F_2 + F_3 & F_3 - 2(F_1 + F_2) & F_2 - 2(F_1 + F_3) \\ * & F_1 + 4F_2 + F_3 & F_1 - 2(F_2 + F_3) \\ * & * & F_1 + F_2 + 4F_3 \end{pmatrix} ,$$

where stars correspond to symmetric coefficients. After elementary linear algebra, the first eigenvalue of $\mathbf{\Lambda}$ can be computed as

$$\lambda_1 = \frac{1}{54} \left(F_1 + F_2 + F_3 + \sqrt{F_1^2 + F_2^2 + F_3^2 - F_1F_2 - F_2F_3 - F_3F_1} \right) .$$

The second eigenvalue is equal to

$$\lambda_2 = \frac{1}{54}\left(F_1 + F_2 + F_3 - \sqrt{F_1^2 + F_2^2 + F_3^2 - F_1F_2 - F_2F_3 - F_3F_1}\right) ,$$

and we have $\mathbf{Tr}(\mathbf{\Lambda}) = \lambda_1 + \lambda_2 = \mathbb{E}[D_{\mathrm{ST}}]/2$. At the cost of less readable formulas, these results could be extended to arbitrary values of c_k, a, b.

References

[1] Wright S. The interpretation of population structure by F-statistics with special regard to systems of mating. Evolution. 1965;19:395-420.

[2] Nei M. Analysis of gene diversity in subdivided populations. Proc Natl Acad Sci USA. 1973; 70:3321-3323.

[3] Nei M, Chesser RK. Estimation of fixation indices and gene diversities. Ann Hum Genet. 1983; 47:253-259.

[4] Patterson N, Price AL, Reich D. Population structure and eigenanalysis. PLoS Genet. 2006;2:e0020190.

[5] Jolliffe I. Principal component analysis. New-York: Springer; 1986.

[6] Bauer FL, Fike CT. Norms and exclusion theorems. Numer Math (Heidelb). 1960; 2:137-141.

[7] Bhatia R. Matrix analysis. New York: Springer Science & Business Media; 2013.

[8] Balding DJ, Nichols RA. A method for quantifying differentiation between populations at multi-allelic loci and its implications for investigating identity and paternity. Genetica. 1995;96:3-12.

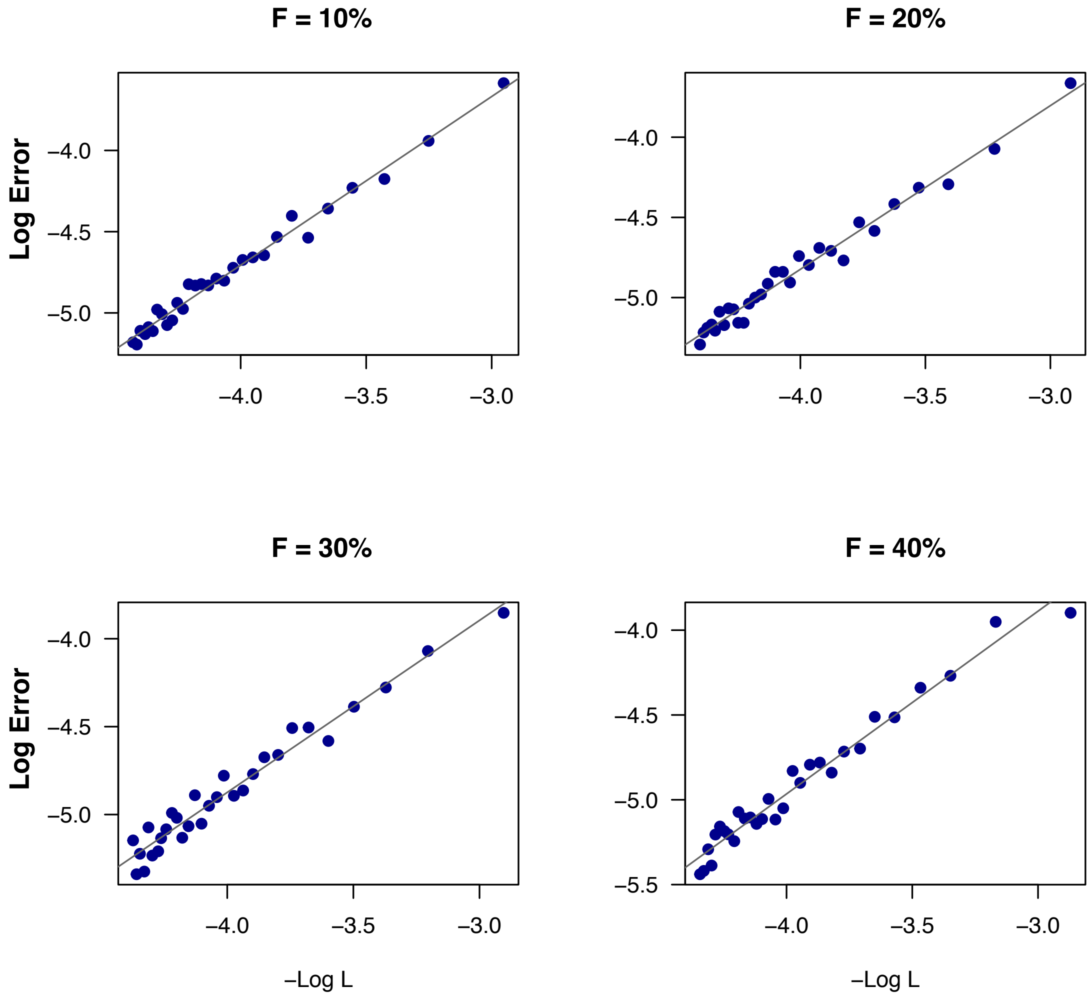

Figure SI A. Order of approximation of $\mathbb{E}[F_{\mathrm{ST}}]$ by PCA leading eigenvalue in two-population models. Difference between the leading eigenvalue and the average value of F_{ST} across loci as a function of the inverse of the number of loci $1/L$ on a log10-scale. Simulations of F-models were performed with p_{anc} drawn from a beta distribution with shape parameters $a = 1$ and $b = 4$, $n = 100$ individuals, and drift coefficients as indicated on the top of each panel.
