# Supplementary material for: A spectral theory for Wright’s inbreeding coefficients and related quantities
Source: PLoS Genet. 2021 Jul 19;17(7):e1009665. doi: 10.1371/journal.pgen.1009665 (PMC8320931; doi:10.1371/journal.pgen.1009665)
Supplement: S1 Table — (PDF) [file pgen.1009665.s002.pdf]

Table S1. F_{ST} **estimates for populations from The 1,000 Genomes Project**

	**Lead. eigen. of PCA***	F_{ST} **across loci**	**Lead. eigen. res. matrix****	**RMT approximation*****
**YRI-IBS**	7.27%	7.27 %	0.31%	0.32%
**YRI-IBS-CHB**	9.75%	9.74 %	0.25%	0.25%
**ACB-ASW**	1.26%	0.60 %	1.05%	0.56%
**PUR-ASW**	3.53%	3.01 %	0.95%	0.56%
**CEU-CLM**	1.40%	1.16 %	0.75%	0.53%
**CEU-MXL**	2.45%	1.86 %	1.06%	0.53%
**CEU-CLM-CHB**	4.77%	4.60 %	0.51%	0.35%
**CLM-IBS-ASW**	4.65%	4.19%	0.52%	0.31%
**ACB-CHB-CEU**	9.00%	8.87%	0.36%	0.34%

* Leading eigenvalue of the PCA
** Leading eigenvalue of the within-population matrix
*** RMT approximation for the leading eigenvalue of the within-population matrix
IBS: Iberian ($n = 147$), **CHB**: Han Chinese in Beijing ($n = 100$), **YRI**: Yoruba ($n = 158$), **CEU**: Utah residents with European ancestry ($n = 104$). **CLM**: Colombians from Medellin Colombia ($n = 102$), **ASW**: Americans of African Ancestry in SW USA ($n = 97$), **PUR**: Puerto Ricans from Puerto Rico ($n = 94$), **MXL**: Individuals of Mexican Ancestry from Los Angeles USA ($n = 100$), **ACB**: African Caribbeans in Barbados ($n = 98$).
