# Supplementary material for: A spectral theory for Wright’s inbreeding coefficients and related quantities
Source: PLoS Genet. 2021 Jul 19;17(7):e1009665. doi: 10.1371/journal.pgen.1009665 (PMC8320931; doi:10.1371/journal.pgen.1009665)
Supplement: S1 Fig — Two simulations of F-models were performed with panc drawn from a beta distribution with shape parameters a = 1 and b = 9, and F = 15%. Top row: n = 200 individuals and L = 69, 248 SNPs. Bottom row: n = 50 individuals and L = 10, 331 SNPs. SFS: Site Frequency Spectrum, MP approximation: Marchenko-Pastur approximation of the distribution of scaled PCA eigenvalues (blue curve). Histograms of scaled PCA eigenvalues representing the proportions of variance explained by the (n − 1) principal axes are displayed in grey color. (PDF) [file pgen.1009665.s003.pdf]

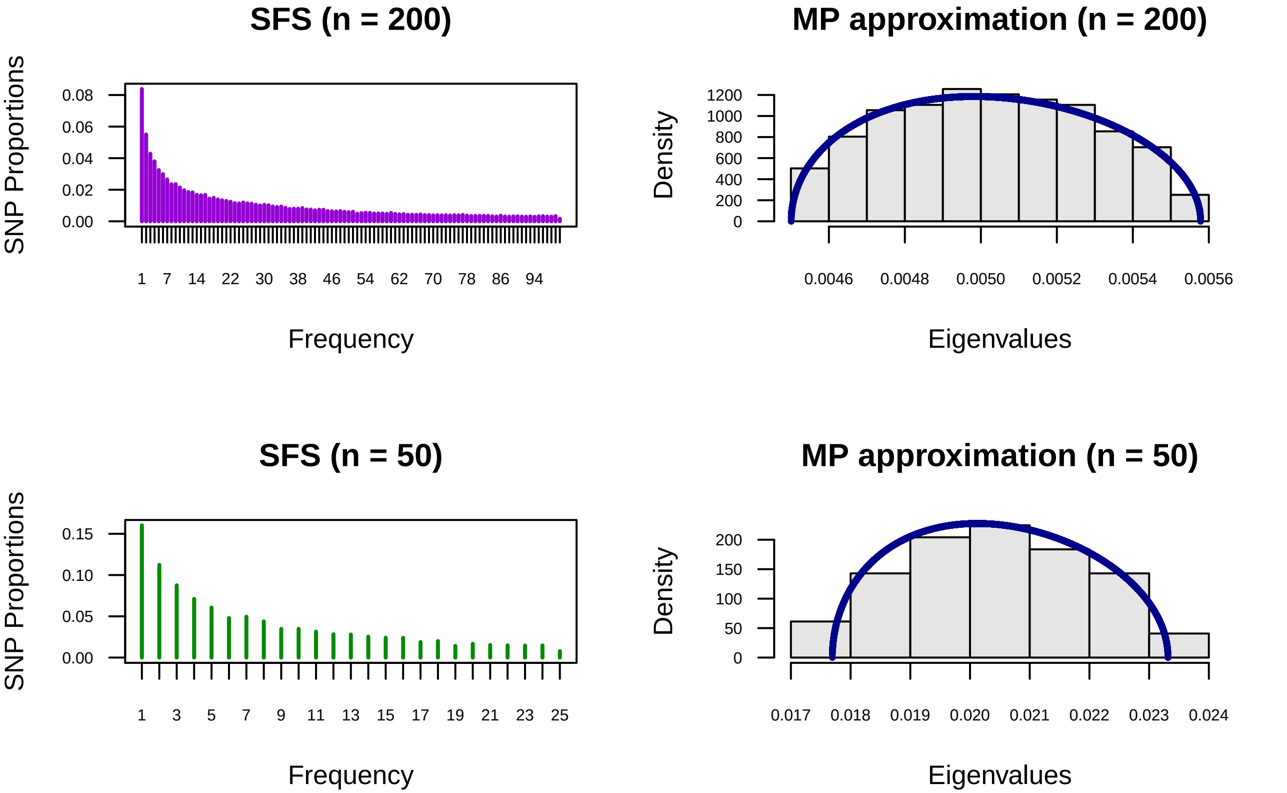
SFS (n = 200)
SNP Proportions
0.08
0.06
0.04
0.02
0.00
1 7 14 22 30 38 46 54 62 70 78 86 94
Frequency
MP approximation (n = 200)
Density
1200
1000
800
600
400
200
0
0.0046 0.0048 0.0050 0.0052 0.0054 0.0056
Eigenvalues
SFS (n = 50)
SNP Proportions
0.15
0.10
0.05
0.00
1 3 5 7 9 11 13 15 17 19 21 23 25
Frequency
MP approximation (n = 50)
Density
200
150
100
50
0
0.017 0.018 0.019 0.020 0.021 0.022 0.023 0.024
Eigenvalues
