# Supplementary figures and images for: A spectral theory for Wright’s inbreeding coefficients and related quantities
Source: PLoS Genet. 2021 Jul 19;17(7):e1009665. doi: 10.1371/journal.pgen.1009665 (PMC8320931; doi:10.1371/journal.pgen.1009665)

**Separation condition (n = 100)**

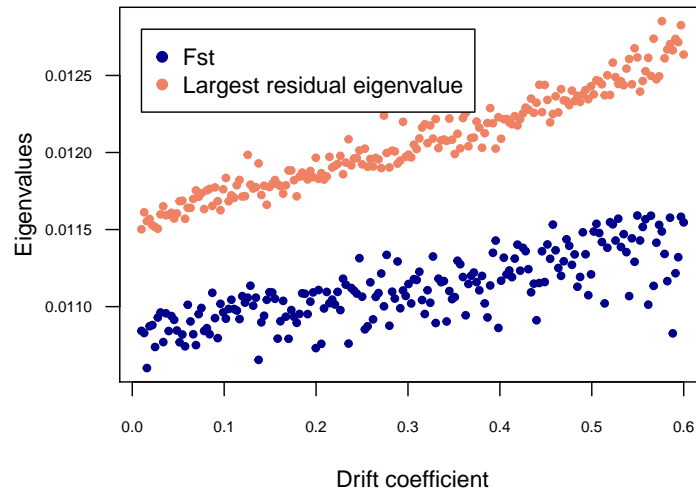

**Separation condition (n = 10)**

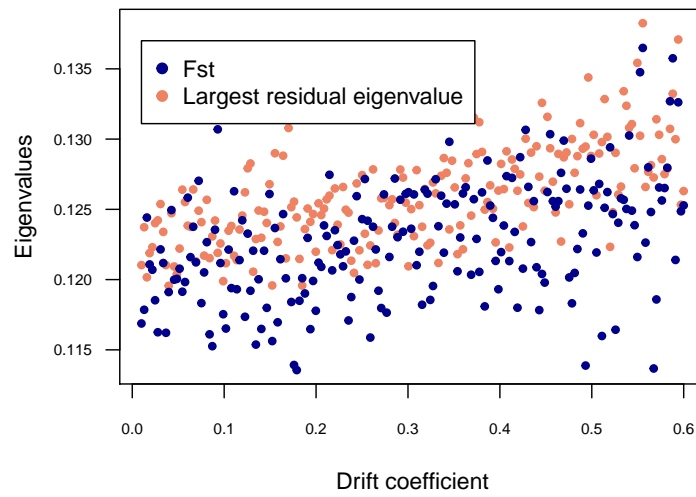

Supplement: S2 Fig — For each value of the drift coefficient, each couple of blue and orange dots represent a simulated data set. FST (blue dots) corresponds to the non-null eigenvalue of the between-population matrix, ZST, and the “residual” value corresponds to the leading eigenvalue of ZS. Population structure is detected when the blue dot is above the orange dot. Top row: 200 simulations with n = 100 individuals and L around 10,000 SNPs. Bottom row: 200 simulations with n = 10 individuals and L around 1,000 SNPs. Simulations were performed with ancestral frequencies, panc, drawn from a beta distribution with shape parameters a = 1 and b = 9. (PDF) [file pgen.1009665.s004.pdf]

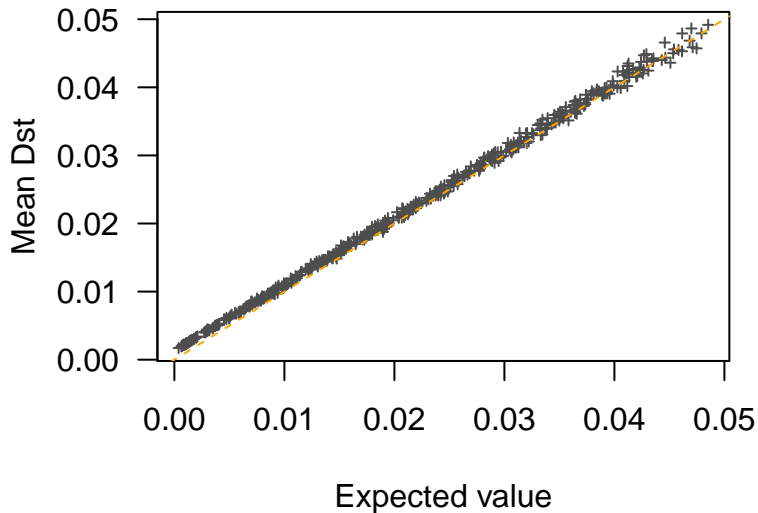

Supplement: S3 Fig — Comparison of values of DST averaged over loci and their theoretical values in F-models. Simulations of F-models were performed with equal drift coefficients (F1 = F2) ranging between 1% and 75%, and sample proportions c1 between 10% and 50% (n = 100 individuals). The ancestral frequencies, panc, were drawn from a beta distribution with shape parameters a = 1 and b = 4. (PDF) [file pgen.1009665.s005.pdf]

**n = 20 - L = 100**

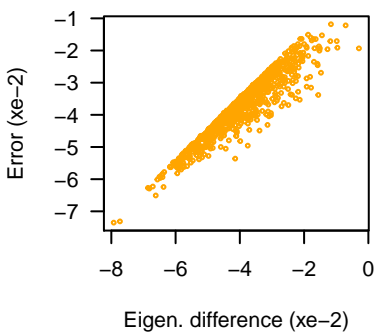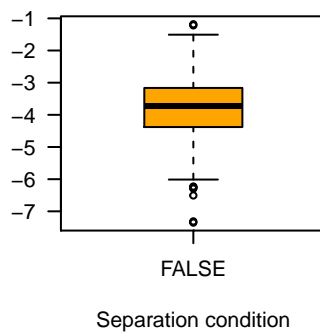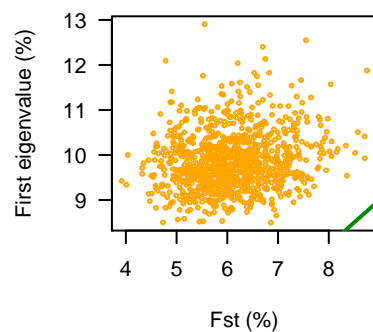

**n = 60 - L = 1000**

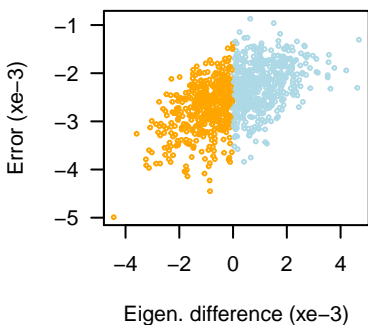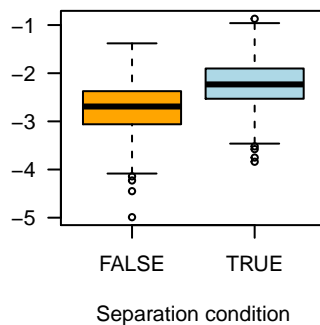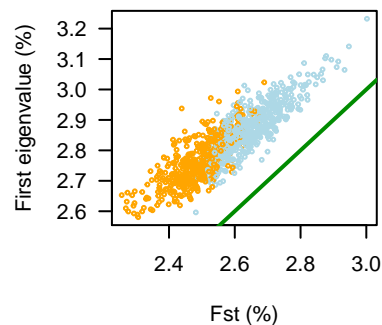

**n = 100 - L = 10000**

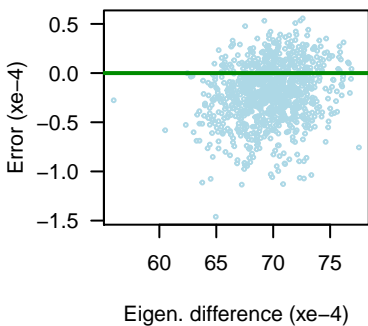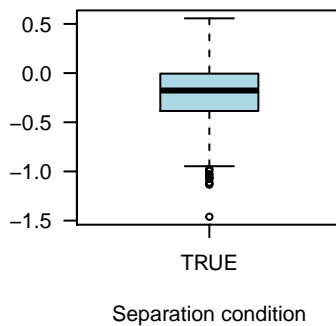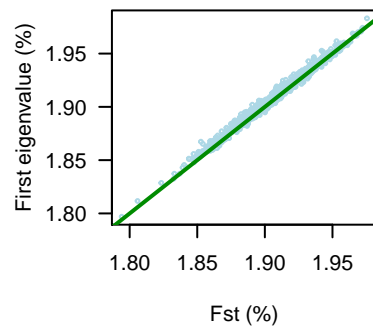

Supplement: S4 Fig — First column: Approximation error defined as the difference between FST and the leading eigenvalue of scaled PCA, E[FST]-ρ12(Zsc)/L, as a function of the difference of eigenvalues, ρ12(ZSTsc)/L-ρ12(ZSsc)/L. Second column: Approximation errors according to whether the separation condition is checked or not. Third column: Leading eigenvalue of scaled PCA as a function of E[FST]. Simulations of F-models were performed for n individuals and L loci with equal drift coefficients F1 = F2 = 0.02. Ancestral frequencies, panc, were drawn from a beta distribution with shape parameters a = 1 and b = 4. (PDF) [file pgen.1009665.s006.pdf]

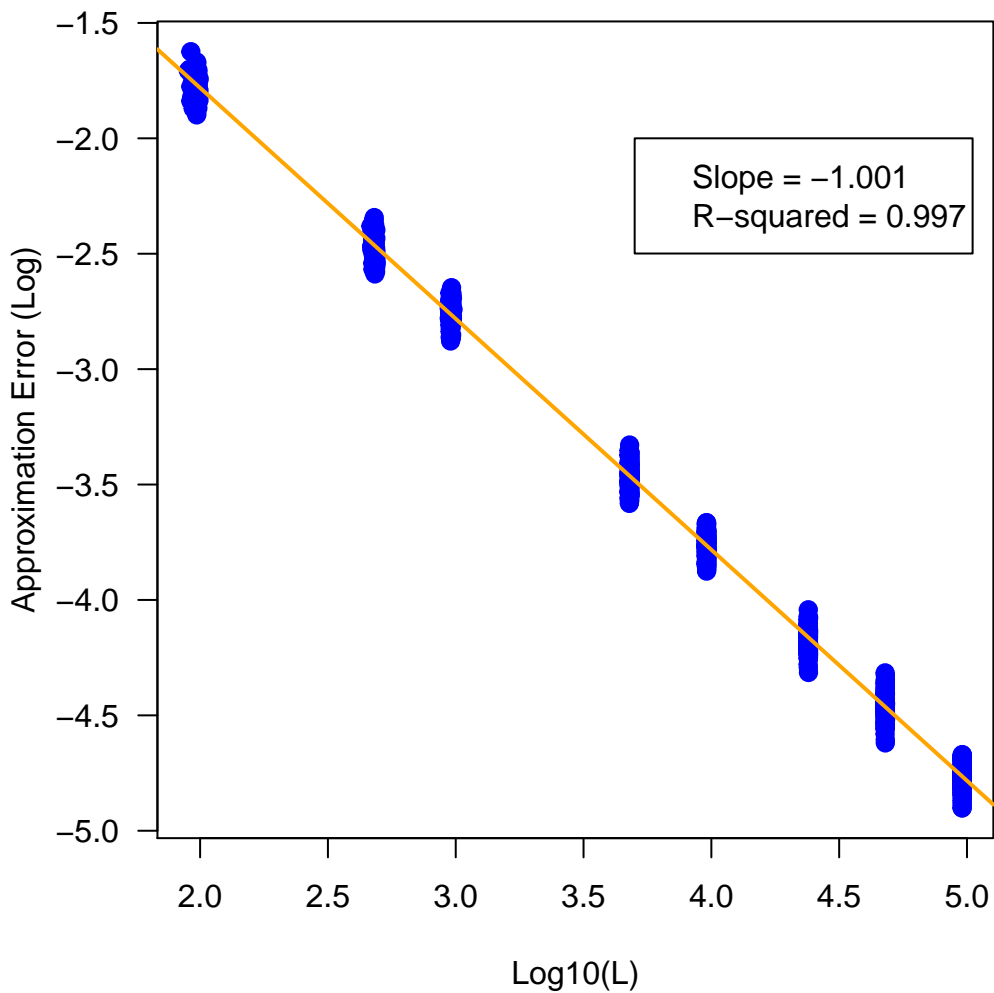

Supplement: S5 Fig — Approximation error defined as the absolute difference between FST and the leading eigenvalue of scaled PCA, E[FST]-ρ12(Zsc)/L as a function of 1/L (L is the number of unlinked loci). The red line corresponds to the linear regression Log(Approx) = a + b Log(L), and has slope equal to b = −1.001 (R2 = 0.997, P < 2e-16). Simulations of F-models were performed for n = 150 individuals with drift coefficients equal to F1 = F2 = 0.02. The ancestral frequencies, panc, were drawn from a beta distribution with shape parameters a = 1 and b = 4. (PDF) [file pgen.1009665.s007.pdf]

A

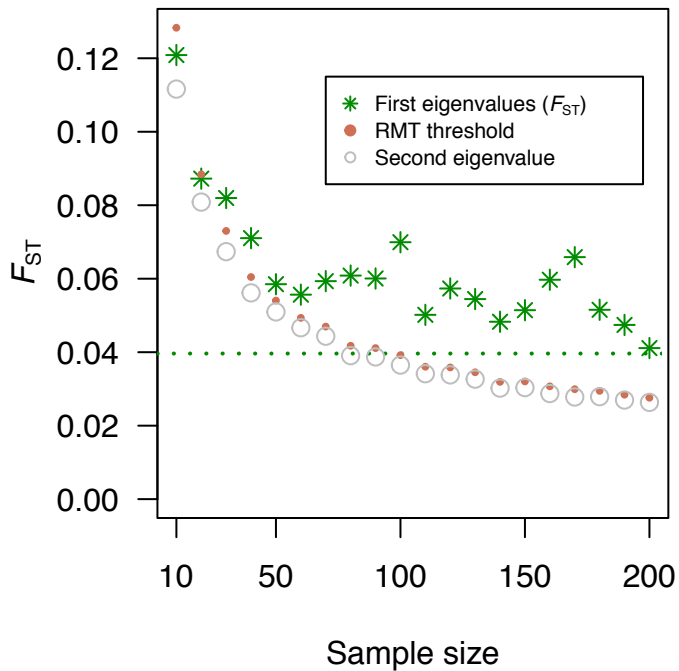

B

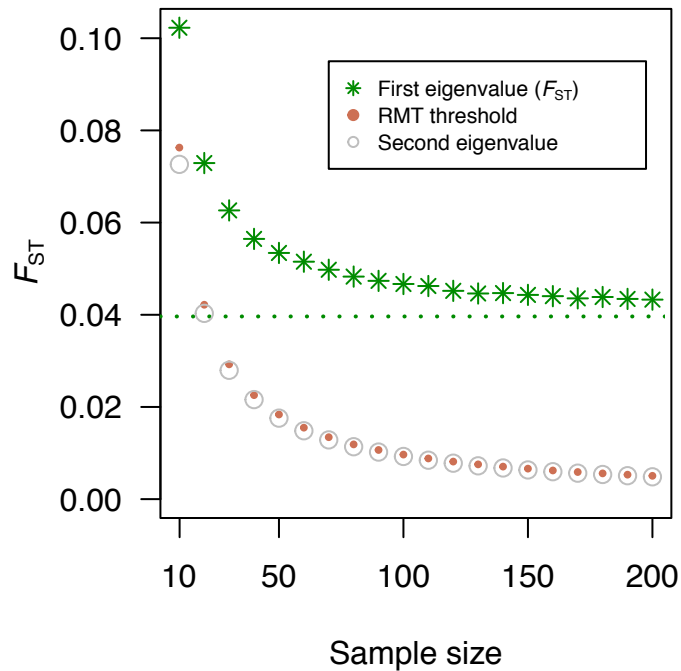

Supplement: S6 Fig — (A) L = 100 loci: The separation condition was verified for sample sizes > 60. (B) L = 100, 000 loci: The separation condition was verified for all sample sizes. FST: Leading eigenvalue of the PCA. RMT threshold: Approximation of the detection threshold from RMT, equal to (1/L+1/n-1)2. Dashed line: Theoretical value for an infinite sample size, E[FST]=3.97%. Simulations of F-models were performed with ancestral frequencies drawn from a beta(1,4) distribution and with F1 = F2 = 10%. (PDF) [file pgen.1009665.s008.pdf]

**A**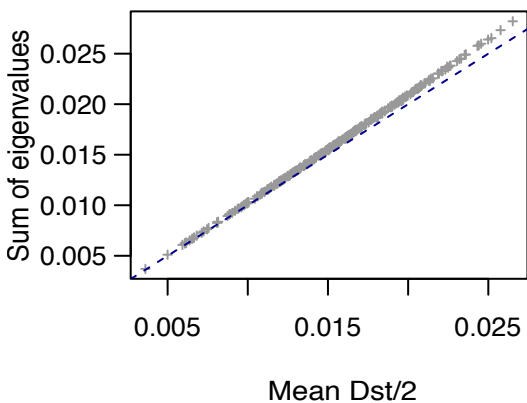**B**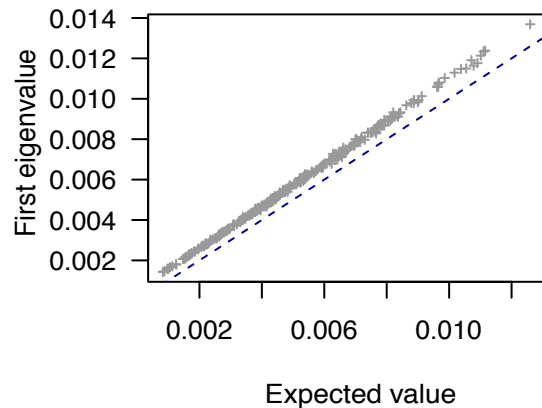**C**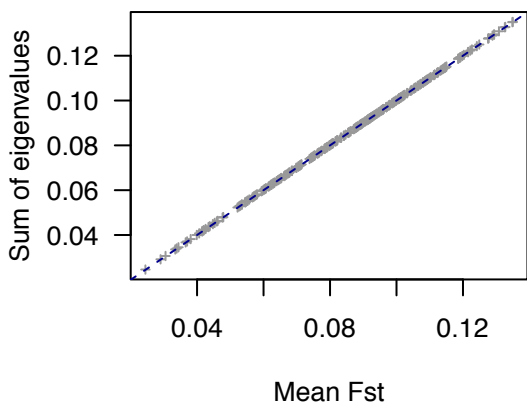**D**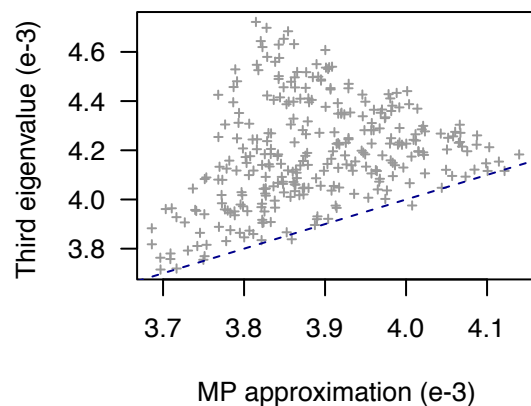

Supplement: S7 Fig — (A) Sum of the first two eigenvalues of centered PCA as a function of the mean of DST/2 across loci. (B) First eigenvalue of centered PCA as a function of its expected value λ1=(F1+F2+F3+F12+F22+F32-F1F2-F2F3-F3F1)/54. (C) Sum of the first two eigenvalues of scaled PCA as a function of the mean of FST across loci. (D) Third eigenvalue of scaled PCA as a function of its approximation from RMT. MP approximation: Marchenko-Pastur approximation of the largest eigenvalue of the residual matrice, ZS/n-3, equal to (1-ρ12-ρ22)×(1/L+1/n-3)2. The dashed lines correspond to the diagonal y = x. Simulations of F-models were performed for n = 100 individuals with drift coefficients F1, F2, F3 between 1% and 25%, equally sampled populations, and ancestral frequencies drawn from the uniform distribution (L = 20000 loci). (PDF) [file pgen.1009665.s009.pdf]

# Probability map

Value

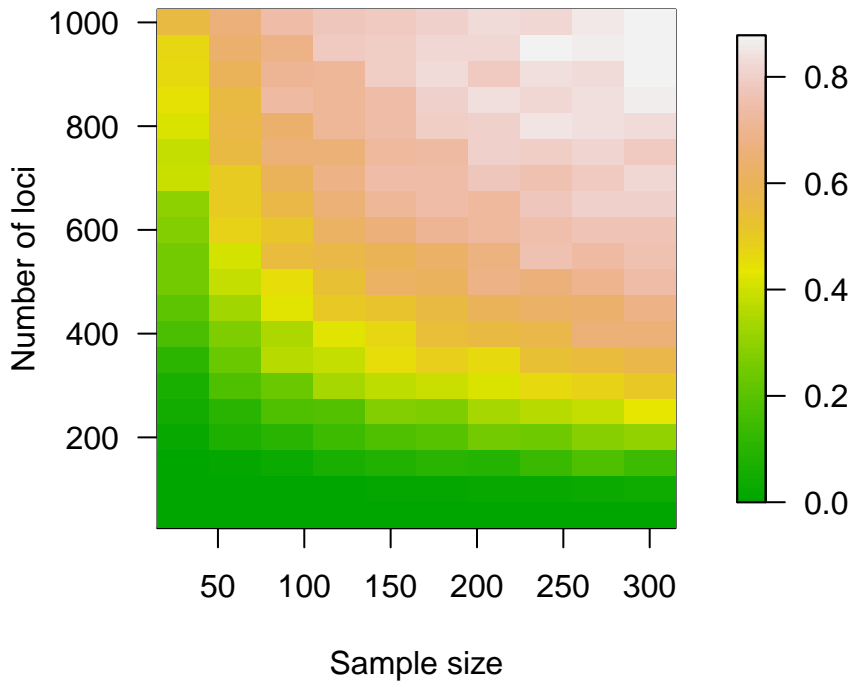

Supplement: S8 Fig — Probability that the separation condition is verified for sample sizes ranging between n = 30 and n = 300 individuals, and number of loci ranging between L = 100 and L = 1000. Simulations of F-models were performed with equal sample sizes, random drift coefficients lower than 10%, and ancestral frequencies drawn from the uniform distribution. Five hundred simulations were performed for each combination of n and L. (PDF) [file pgen.1009665.s010.pdf]

**A**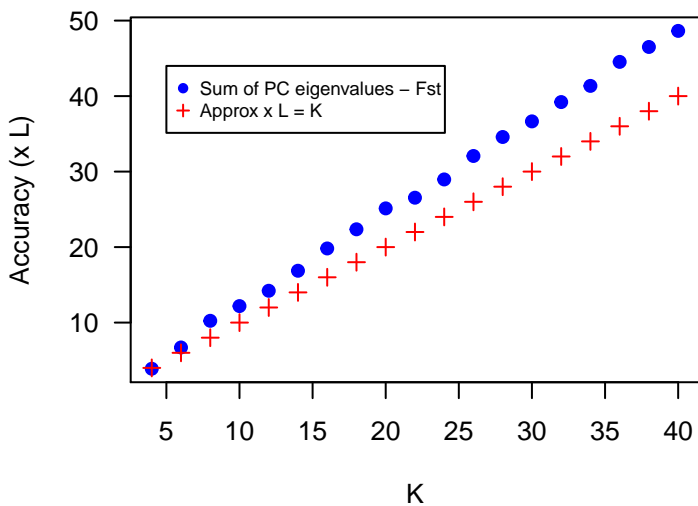**B**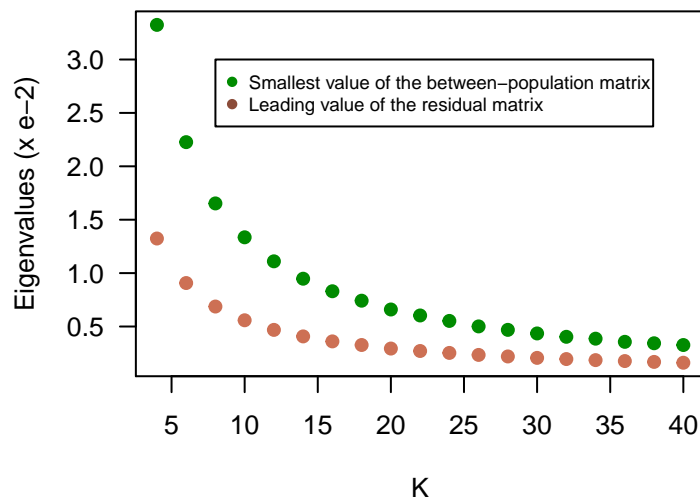**C**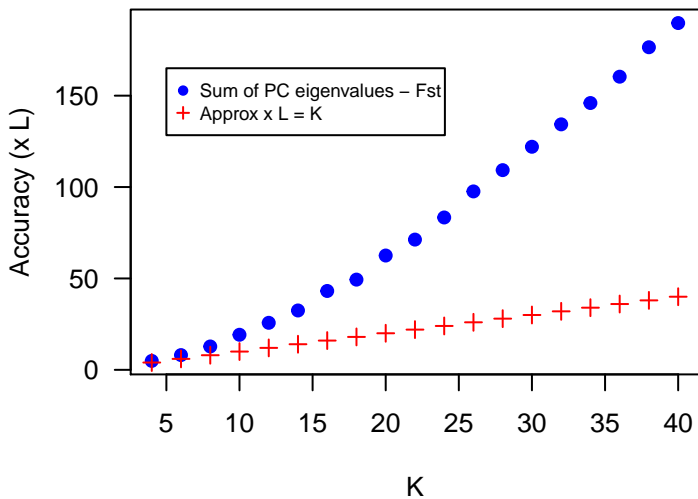**D**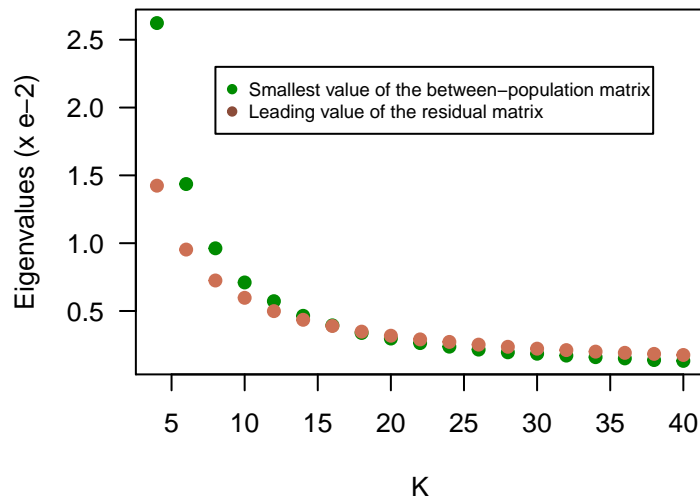

Supplement: S9 Fig — A-B) Simulations with equal drift coefficients Fk = 0.1. The accuracy of the approximation of E[FST] by the sum of the K − 1 leading eigenvalues of the PCA is comparable to K/L (A) and the separation of eigenvalues from the residual matrix is verified (B). C-D) Simulations with unequal drift coefficients Fk = 0.2/k. The accuracy of the approximation of E[FST] diverged from K/L when the separation of eigenvalues did not hold. In all simulations, ancestral frequencies were drawn from a beta distribution with shape parameters a = 1 and b = 4. Sub-population samples had size equal to nk = 20, the total population size was n = 20 × K), and the number of SNP loci was L ≈ 19850. (PDF) [file pgen.1009665.s011.pdf]

# Ancestry matrix

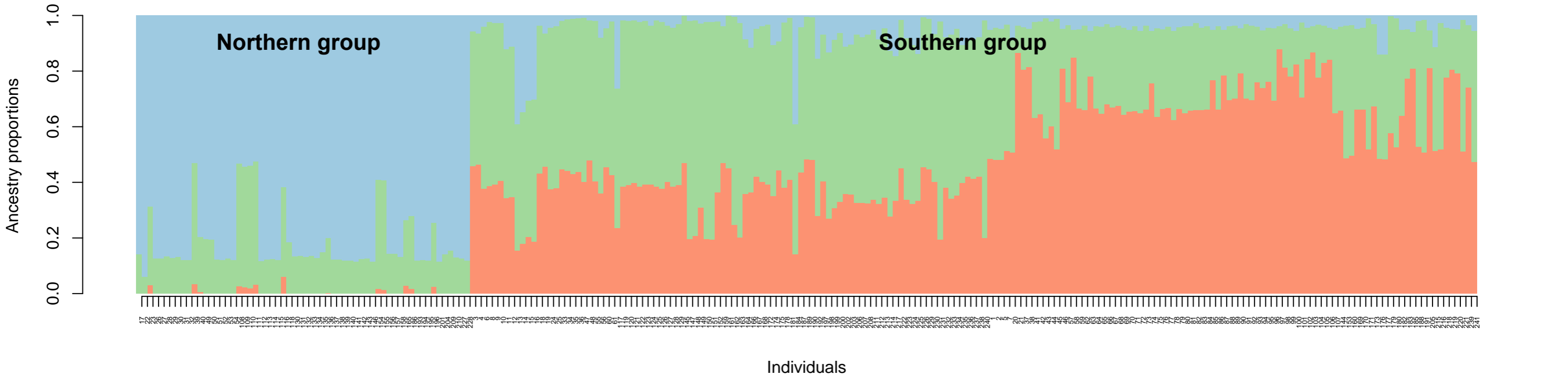

Supplement: S10 Fig — Ancestry coefficients obtained from the spatially explicit ancestry estimation program tess3r with K = 3 populations. The southern group exhibits substantial levels of mixed ancestry. (PDF) [file pgen.1009665.s012.pdf]
